# The Diseases, Etc., of the Bible

**Published:** 1856-07

**Authors:** W. T. Grant

**Affiliations:** Wrightsboro’, Columbia county, Ga.


ATLANTA

Medical and Surgical Journal.

VOL. I.] JULY, 1856. [NO. 11.

### ORIGINAL COMMUNICATIONS.

### ARTICLE I.

*The Diseases, etc. of the Bible.* By Dr. W. T. GRANT, of Wrightsboro', Columbia county, Ga.

I am not aware that any one has ever attempted an Essay on this subject before. It is therefore a *terra incognita*, an unknown region, I have set myself to explore. And in its investigation, I have not the usual facilities offered by a multiplicity of authors of reference, of which we may avail ourselves in an investigation of any other part of Medicine; but I am left entirely alone to gather together the scattered and disjointed fragments of medicine, which are interspersed all through the writings of the Biblical authors. Nor is this all the difficulty connected with the subject—after collecting together all the subject-matter which is mentioned in the Bible, it is then necessary to determine the nature and character of a large number of diseases of which we have the most meagre and indefinite details; and also to arrange and classify them with some of our modern diseases. In all this I have no professional assistance from any quarter whatever. This must be my apology, therefore, for whatever errors I may make in the investigation.

We are not able at this day, to determine when, where, or how disease first began. We know that it has existed for ages; but of its origin we know nothing. I think, however, that we may safely take the position that disease was almost coeval with man, and I think it could be well sustained by reasoning upon the cognate sciences of Botany, Geology, and Chemistry. Botony tells us that the vegetable kingdom of the present day is analogous to that of the primitive ages; Geology informs us that the same changes which are now going on in the texture of our planet, have been going on ever since the birth of man; and Chemistry tells us that the original elements of matter, as known to us, are the same original ultimate elements out of which the Great Architect of the universe fabricated our world, and "all therein contained." They were endowed with the same properties and exhibited the same play of affinities, that we see possessed by those of our day. Hence, I conclude that the same causes which are in operation to-day, the same changes which ensue, and the same effects which result therefrom, were all likewise in operation on the day of Adam's creation. From this we make a very easy transit to the conclusion that disease existed, or at least that its cause was in existence, so early as Adam's time. There may not have been cases of disease at that immediate time, for there was not the human material for the *materies morbi* to act upon.

The credible records of medicine do not extend back more than three or four centuries, and cannot therefore throw any light upon this subject. And within that time there has not *originated* a single *sui generis* disease. All the diseases to which "flesh is heir" have been transmitted to us from our ancestors of olden times. And it may be said that the East was the cradle of disease as well as the cradle of mankind. We know that we have a great many diseases among us, of which we can give no account—we know not whence they came. This is particularly the case with all our contagious diseases, the history of which is lost in the obscurity of ages. Who can determine the date of the first case of Variola, or of Scarlatina, or of Rubeola? The Small Pox was known in Arabia nearly a thousand years ago, and was supposed by Rhazes and other Arabian Physicians, to have been introduced into that country from Ethiopia; and it is supposed to have visited Europe as early as the seventh century. Nor is this observation confined to this class of diseases alone. If we will go beyond the limits of the Bible and examine into the evidence furnished by the profane writers, we find an innumerable list of diseases, of the nature of which we can form no conjecture from the imperfect account we have of them. I think we may safely conclude that diseases—perhaps different from ours, perhaps not—were as prevalent among the nations of old as they are at this day. But the limits of my subject will not permit me to do this. It would be an interesting subject for investigation—the ancient history of Medicine—and one which I may take occasion to investigate some time—but cannot do it now. It will suffice for the present, however, to know that the existence of a great many diseases will be made manifest, independently of those mentioned in the Bible, by the frequent reference which it is necessary for me to make to profane historians, either for the purposes of elucidation or of supporting conclusions deduced in this essay. I have found about fifty different diseases mentioned in the Bible, beside a reference to the existence of many others. The first mention of any disease is found in 12th Genesis, where it is stated that Pharaoh and his house were plagued with great plagues; this plague was visited upon Pharaoh about 1900 years B. C., and consequently about 3750 years ago. Of these fifty different diseases, (among which, for convenience, I have classed Menses, Labor, Sterility, &c.,) I shall attempt a description of but few, because the others are merely mentioned, and not described. Biblical Pathology is very meagre, although Biblical Nosology is sufficiently extensive. The number of *cases* of diseases, including Medical, Surgical and Obstetrical cases, which I find recorded in the Bible, amount to *sixty-eight*, and are as follows: Of Leprosy 21 cases, of Menses 2, of Premature Labor 2, of Sterility 7, of the Bloody Issue 1, of Deformities 1, of Injuries 1, Ulcers, Boils or Blains 4, Mortification 2, Paralysis 1, Tonguetie, or Anhyloglossum 1, Lunacy 1, Apoplexy 1, Epilepsy, 1, Fever 2, Dysintery 1, Dropsy 1, unknown disease 2, Blindness 8, Deafness 1, Dumbness 1, Dumbness and Blindness combined 1, Serpent's bite 1, and of that affection spoken of as being "possessed of a devil" 4.

I will now proceed to discuss, in detail, all the medical matter which I find recorded in the Bible.

*Physicians.*—Physicians are, so far as I have been able to ascertain, spoken of four times in the Bible. The first time in Genesis 50, second verse, nearly seventeen hundred years B. C., in the following language: "And Joseph commanded his servants, the Physicians, to embalm his father: and the Physicians embalmed Israel." In 2nd Chronicles, xvi, 12, about 1,000 years B. C., Physicians are again spoken of.—Also, we find them mentioned in Jeremiah viii, 22, 600 years B. C. And Luke, who was himself a Physician, mentions them in the following connection, in his viii chapter, 43d verse: "And a certain woman having an Issue of Blood twelve years, who had spent all her living upon Physicians, neither could be healed of any." In each of these references we see that there is mentioned more than one Physician, so I think that we may conclude that Medicine was pursued as a regular profession even at that early day. This conclusion is supported by reference to those historians who wrote in those early times. Tacitus mentions several Physicians, and also speaks of "the practice of Physic." Herodotus who wrote about 450 years B. C. also mentions a large number. He uses the following language: "Damacedes went to Egina—he excelled the most skillful of the Medical profession." He says also that there were no Physicians in Babylon, and that those of Crotono, excelled those of Cyrene. The following is from Herodotus: "The art of Medicine in Egypt is thus exercised: one Physician is confined to the study and management of one disease; there are of course a great number who practice this art; some attend to disorders of the eyes, others to those of the head; some take care of the teeth, others are conversant with all diseases of the bowels, whilst many attend to the cure of maladies which are less conspicuous." I need not extend this reference—this is sufficient. I could quote more largely both from Tacitus and Herodotus, as well also as from Josephus; but the above will answer my purpose, and I cannot trespass further beyond the limits of my subject.

*Emerods.*—The only connected account that we have of this disease, so far as I have been able to ascertain, is contained in the fifth chapter of 1st Samuel. The Philistines had beaten the Isralites in the battle of Ebenezer, and had taken as one of the trophies of war, the ark of the covenant. They first carried it to the city of Ashod, among the inhabitants of which city this disease soon made its appearance. They then removed the ark to Gath, where also the disease broke out. Its character in this last city, is somewhat analogous to the Venereal, for it is said that the inhabitants were smitten "with the Emerods in their secret parts." From Gath the ark was sent to Ekron, where also the disease broke out. It also visited Gaza and Askalon. The account of this epidemic that Josephus gives is as follows: It began in the city of Ashod, and spread thence to Ekron, Gath, Gaza, and Askalon; these five cities being the only ones spoken of as being visited by the distemper. Josephus calls it a "dysentery or flux," and says that it was "a sore distemper that brought death upon them very suddenly; for before the soul could, as usual in easy deaths, be well loosed from the body, they brought up their entrails, and vomited up what they had eaten, which was entirely corrupted by the disease. This is a very good description of the cholera. And the fact that Judea is not a great distance from, and is in nearly the same climate and latitude with India, the hot-bed of cholera, will lend some support to the opinion that the above epidemic of the Emerods corresponds with the Cholera. About the year 1830, at the time when the Asiatic Cholera was making its way westward, it branched in Syria, one branch pursuing its way down through Syria and the adjoining countries into Arabia and Egypt. This was, without doubt, the first time it ever visited Europe; but will any one say that it had never before invaded Judea and Western Asia? I consider this opinion as altogether problamatical, but I offer it for what it is worth.

*Leprosy.*—I know of but two countries in which this disease (*positive* Leprosy,) is prevalent at the present time, Palestine and Mexico. The inhabitants of the former do not consider the Leprosy of our days as the same that existed in olden times—but I do not think this opinion worth much, and shall therefore extract the following description of the disease from Kendall's Santa Fee Expedition, vol. II. He was confined for some time in the Hospital of San Lazaro (Hospital for Lepers,) in the city of Mexico, and is therefore competent to give a good description of the disease. He says that at the time of his confinement in the above Hospital, there were fifty or sixty males, and a greater number of female lepers in it. He says of the disease: "It makes its first appearance by scaly eruptions on different parts of the face and body of the victim, and these eruptions are never perfectly healed. The limbs of many, and more especially the hands, at first appear to be drawn and twisted out of all shape. Gradually the nose and parts of the face are carried away, while the features become distorted and hideous. The voice assumes, at times, a husky and unnatural tone, and again the doomed patient is unable to articulate except in a shrill piping treble. With many, when near the last stages, all powers of speech are lost, and vainly do they endeavor to make known their wants, by sounds which belong not to this earth of ours." This description accords with the disease as we find it recorded in the Bible, in many particulars. Kendall says in the context, that he saw numbers who had entirely lost their nose, as well also as a great many who had entirely lost their fingers. The Biblical description of the disease is as follows: It begins as a rising, scab or bright spot beneath the skin, generally of the face, although any part of the body may be attached from the beginning. If the eruption begins in a part of the body covered with hair, as the scalp, pubes, or on the chin in men, the disease turns the hair white; or if there be any hair on the part where the disease begins, as on the legs or the back part of the fore-arm, the hair is turned white. It may begin in one or many spots, and its tendency is to destruction and disorganizing ulceration—never to a healthy termination. It would seem, from the accounts we have, that Leprosy was not considered contagious, for we find a number of instances recorded, both in the Old and New Testament, of Lepers who were permitted to go at large. Its invasion was occasionally sudden as we find it in the case of Uzziah the king (2nd chron. xxvi, 16,) Gehazi (2nd kings v, 27,) and of Miriam (Numbers xii, 10.)

According to the Mosaic code the diagnosis of Leprosy was easy and plain. If the spots were white and beneath the skin, and if there was a hair on the spot, and the hair was white also, it was plainly Leprosy. These conditions were considered pathognomonic of the disease.

Some theologians speak of Leprosy as a disease that begins inwardly—within the system, and may remain latent for a great length of time. There is no doubt in my mind that the disease is constitutional, and that the eruption is but the local manifestation of the constitutional disorder. I will take occasion here to remark, that whenever any disease, whatever may be its character, is capable of manifesting itself locally in two or more separate and distinct parts of the system, that such disease is constitutional in its nature, and that its treatment should be regulated accordingly.

Some of the profession in England, among others Erasmus Wilson, have supposed that Leprosy and Elephantiasis were the same disease. Indeed, there seems to be a great inclination in the profession generally to take this position. This seems unaccountable to me—and I consider the opinion as wholly gratuitous, and cannot understand how the same name or term can be applied to two diseases whose pathology are different. I do not deny that Leprosy and Elephantiasis may be kindred diseases—so are Small Pox and Scarlet Fever; but that is no reason that they should be considered one and the same disease, when their pathology are so different.

The only treatment mentioned in the Bible for Leprosy, is that recommended by Elisha to Naaman—to wash himself seven times in the river Jordan, which he did and was cured. I have found mention made of 21 cases of Leprosy in the Bible. Luke, speaking on this point, says: "And many lepers were in Israel, in the time of Eliseus the prophet."

*The Plague or Pestilence.*—The accounts which we have of the Plague are very scanty. It is true that the disease is mentioned often enough, but the mention made of it is not sufficiently definite to enable us to arrive at any positive conclusion as to the nature of the pestilence. The presumption is, that the term plague or pestilence is generic, and was used by the Biblical writers to signify any wide-spread or malignant Epidemic. This is the meaning attached to it by Josephus. He describes the Plague, mentioned in the last chapter of 2d Samuel, which befell the Israelites, soon after the numbering of the people by David, contrary to the command of the prophets. Nor can we form any opinion as to the nature and character of the particular disease which constituted that Plague. The description by Josephus is as follows: "God sent a pestilence and a mortality upon the Hebrews; nor did they die after one and the same manner; nor so that it was to know what the distemper was. Now, the miserable disease was one indeed, but it carried them off by ten thousand causes and occasions, which those that were afflicted could not understand; for one died upon the wreck of another, and the terrible malady seized them before they were aware and brought them to their end suddenly; some giving up the ghost immediately with great pains and bitter grief, and some were worn away by their distemper, and had nothing remaining to be buried, but as soon as ever they fell were entirely macerated; some were choked, and greatly lamented their case, as being also stricken with a sudden darkness; some there were who, as they were burying a relative, fell down dead, without finishing the the rites of the funeral." Josephus states that seventy thousand perished of this plague in one morning. We have no reason to doubt the correctness of Josephus' description of this Plague; but wemust not fall into the error of considering this a good description of every Plague mentioned in the Biblical records; for as before remarked, the term Plague or Pestilence was used generically to signify any exceedingly fatal epidemic, and that the disease here described was but one phase or form of Plague. This Plague as described by Josephus (and it is the only described Plague that I have been able to find) is very anamolous and corresponds with no known disease of the present day. The rapidity of its progress in some cases—the slow consumption of the body in others, and withal its exceeding fatality, mark it as a disease separate and distinct from any and all the diseases known to the moderns.

*David's Affliction.*—David's affliction was two-fold in its nature—mental and physical. His mental disease was undoubtedly Hypochondriasis. There is a melancholy tone pervading all the Psalms, which would lead any one to suppose that David, the author, was of a most melancholy disposition, and always mentally troubled in medical language—that he was a confirmed Hypochordinac. His continually bewailing his fate, his speaking of the hypocrisy of mankind generally, and of his own immediate acquaintances, particularly of the loss of his friends, and of their scorning and mocking him—all proves to my mind that he was of an exceedingly melancholy disposition, and that he viewed the world at large, as well as his own most intimate friends, through the medium of a highly excited and morbid fancy. He speaks of himself as being forsaken both of God and man. I do not believe that he was so entirely forsaken, but believe that his thoughts were dictated by a diseased imagination, and that he was afflicted with that mental malady known to us as Hypochondriasis. His physical disease may have induced his melancholy, for physical disease frequently brings about this state of things, as every practitioner of medicine will have noticed. Still, although we have a coincidental existence of bodily disease and mental suffering, in David's case, yet this will not bear us out in the positive assertion that his melancholy was the result of his physical affliction. We may presume, however, that this was the case, but this is as far as the facts of the case will entitle us to go in our inferences.

David's physical disease is very fully described, but in this place I shall mention only a part of his affliction, reserving the rest to be brought forward in another part of this essay. It consisted in part of an entire loss of all the fat of his system; he was greatly weakened, but himself ascribes this to abstinence. His loss of flesh, as it is commonly called, was so great that his bones clave to his skin. And in his old age his system had so far lost its tone that it could not generate a sufficiency of heat to keep him even comfortable, although they "covered him with clothes." In consequence of which, it became necessary to procure a young person to lie with him, to impart the *quantum sufficit* of heat to him necessary to keep him warm.

### MIDWIFERY.

*Midwives.*—The first mention made of midwives, is in Exodus i, 15, and from the phraseology of the verse we might conclude that during those ancient times the office of Midwife was performed by two or more persons, according to the population of the nation or people over which they presided in that capacity. The writer of Exodus says that Pharaoh "spoke to the Hebrew midwives of whom there were *two*, Shiphrah and Puah." This verse most evidently means that there were but two Hebrew midwives. If it had been written thus, Pharaoh "spoke to the Hebrew midwives Shiphrah and Puah"—then it would imply, that there were a number of Hebrew midwives, but that Pharaoh wanted the services of but two of them, Shiphrah and Puah. But as it is, it most evidently means that there were at that time but two Hebrew midwives. The following from Josephus gives support to this inference. He says that Pharaoh directed that "the Egyptian midwives should watch the labors of the Hebrew women, and observe what is born, for these were the women who were enjoined to do the office of midwives to them; and by reason of their relation to the king would not transgress his commands." The latter part of this quotation is that to which I would call attention, as giving support to the above opinion.

*Menses.*—This physiological action of the ovaries was of course as common among the Israelites and other ancient nations, as it is among us of the present day. I find but two cases, however, mentioned in the Bible—that of Sarah, and that of Rachel.

*Flooding.*—It seems to me that flooding is intended to be expressed in Leviticus xv, 25, a part of which verse I will quote: "And if a woman have an issue of her blood many days out of the time of her separation, or if it run beyond the time of her separation." Now, if the expression—"the time of her separation" refers to labor, then it is evident that flooding is referred to. The expression may mean the menstrual period. There is no positive evidence to influence either conclusion; but I am inclined to think that it refers to labor, from the fact that in every instance in the Bible, when the menstrual period is intended to be expressed, it is spoken of either as the "menstruous period," or as "the custom or habit of women."

*Premature Labor.*—The expressions, "an hidden untimely birth," "a mis-carrying womb," "the untimely birth of a woman," "an untimely birth," &c., occurring in the books of Job, Hosea, Psalms and Eclesiastes, show that premature labor was well known to the ancients, although it may not have been as common as at the present day. Job speaks of it about 1520 years B, C., and the author of Ecclesiastes speaks of it about 977 years B. C. It would appear that it was not uncommon in those days to produce abortion, from the following from Josephus: "The law enjoins us to bring up all our offspring, and forbids women to cause abortion of what is begotten, or to destroy it afterwards." I find the following in Exodus xxi, in reference to the same thing—"If men strive, and hurt a woman with child, so that her fruit depart from her, and yet no mischief follow, he shall be surely punished," &c. This quotation shows that abortion was sometimes brought on and gotten safely through with by the mother. Further on, in the same chapter, it is spoken of as being followed by mischief, meaning probably the death of the mother.

(I will introduce the following from Tacitus: "Nero issued a proclamation declaring the guilt of Octavia. * * * He added, that by the use of medicines to procure abortion," &c.)

I find two cases of premature labor mentioned in the Bible —the one occurring in the wife of Phinebas, the other in Jacob's wife, Rachel. I shall speak of them somewhat in detail. Phinebas' wife had heard of the deaths of her father and husband, and the loss of the ark of the covenant, and was, by this news, brought to premature labor, by which she lost her life. Josephus says that this labor occurred at the the seventh month. Icabod, who was born at the time, lived. According to Biblical chronology this premature labor occured about 1140 years B. C. The second case—that of Rachel-- is found in Gen. xxxv. It appears that as Jacob was journeying, with all his houshold, from Bethel to Ephrath, his wife, Rachel, being with child, was taken in labor and lost her life by it. It is said that she "had hard labor." I think that the probabilities are in favor of this case being one of premature labor, for it is not at all probable that Jacob would have still continued his journeying, if his favorite wife, Rachel, was so near to confinement, as the circumstances of the case prove her to have been, if it had been labor at full period. The mode of travel of those days was such that it was very dangerous for a woman advanced in pregnancy to go on a journey; there were no carriages of any kind in use in those days, so far as we know, and all journeys were performed either on foot or on the back of some animal. This case of premature labor occurred about 1730 years B. C. Benjamin was Rachel's son by this labor.

*Sterility.*—I find an account of seven cases of sterility, only one of which was permanent, that of Michal, the daughter of Saul; her sterility continued during her life. The following are the cases in which the sterility lasted only for a time: Sarah, Abraham's wife, (Gen. xvi: 1;) Leah and Rachel, Jacob's wives, (Gen., xxix: 31;) Manvah's wife, (Judges, xiii: 2;) Hanah, (1 Sam., i: 5;) and Elizabeth, (Luke, i: 7.) If we take the marginal reading of 2 Kings, ii: 19, 21 verses, we learn that it was supposed, about nine hundred years B. C., that the waters of Jericho were liable to produce sterility, and that Elisha remedied this quality of the waters by throwing a quantity of salt into them.

*Bloody Issue.*—This expression is found both in the Old and New Testament; and there seem to be two meanings attached to it. The one, is the birth of a child; the other, a real issue of blood—a true hemorrhage. We find the first meaning embodied in the 13th chapter of Leviticus. I will give a synoptical statement of this chapter. If a woman conceives and bears a male child, she shall be unclean for seven days, "and shall continue in the blood of her purifying thirty-three days." If it is a female child, she is unclean 14 days, "and shall continue in the blood of her purifying sixteen days." And at the end of her days of purifying, she shall bring a certain burnt-offering and a certain sin-offering, according as the child is male or female. And the first shall make an atonement, and "she shall *be cleansed of the issue of her blood.*" I will make but one comment upon this chapter; and that is, that the evidence contained in it in support of my conclusion, is entirely negative in its character. The issue of blood spoken of must mean the birth of the child, because it cannot, in its present connection, mean any thing else.

The other meaning attached to the expression is found in 15th Leviticus, 9th Matthew, and 8th Luke, and clearly signifies a hemorrhage, as we see by the following, from the 15th of Leviticus: "If a woman have an issue, and her issue in her flesh be blood." This must mean an issue of blood from one or more of the natural cavities of the body, and through some one or more of the natural outlets of the body, and could not signify a hemorrhage from a wound, for wounds never rendered a person unclean, whilst the issue of blood always did. There is recorded, both in Matthew and Luke, the case of a woman who had an issue of blood during twelve years. I should consider this case as one of atonic hemorrhage, in which there was a slow and continuous dribbling away of the blood. The idea that it was a continuous flow of blood, is conveyed in the expression—"and immediately her issue of blood staunched." She was then bleeding at the time of her cure, and had had this issue for twelve years.

The next thing in connection with this subject, is to determine the identity of the hemorrhage. The term, "issue of blood," when severed from its connections, may mean hemoptysis, hematemesis, hematuria, menorrhagia, or any other hemorrhage. I think that in every instance in which it is used in the Bible, with the exception of the 13th chapter of Leviticus, it means a hemorrhage from the uterus, or some of its appendages; for there is no instance in all the Bible in which it is mentioned, except in connection with a woman. An issue of blood from a man is never spoken of; it is always from a woman. Now, in regard to the hemorrhages in general, males are as liable to them as females; there is no difference so far as I know, between the sexes in their relative liability to hemorrhage. But there is one hemorrhage, which, from her peculiarity of organization, is confined exclusively to woman; and that is menorrhagia. Now, this fact, that woman alone is subject to this particular hemorrhage, taken in connection with the fact, that the "issue of blood" is always spoken of in the Bible, in connection with women, and never in connection with men, will, I think, go far to identify the "issue of blood" of the Bible, with the menorrhagia of obstetritians.

### SURGERY.

*Roller Bandage.*—I find mention made once only of the roller bandage, in Ezekiel, 30-31. The verse containing the mention is metaphorical, and to the effect, that the arm of Egypt was broken. Then follows the passage, "it shall not be bound up to be healed, to put a roller to bind it." This is recorded 512 years B. C., and proves that the roller bandage was known and in use at that period of time. I am told that when Geo. R. Gliddon, Esq., opened an Egyptian mummy in Boston, some years since, he found enveloping it, nearly all, if not quite all, the different bandages known to modern surgery. Herodotus mentions the case of one Pythes, who was severely wounded, and taken prisoner by the Persians. "They made every possible effort to preserve him, bathing his wounds with myrrh, and applying to them *bandages of cotton.*"

*Circumcision.*—The operation of circumcision was established by Moses among the Israelites, and is first mentioned in 17th Genesis. The special exact operation is described neither in the Bible, nor in Josephus, but I suppose it to have been the same that is performed at this day.

*Deformities.*—There are several deformities mentioned in the Bible, as we see by the expressions "lameness," "crook-backed," "blemish in the eye," &c.; but we have very little account of them other than the simple mention made of them. I find account of two congenital deformities, the one in Acts 3; the other in Acts 14. There is a case of lameness mentioned in 2 Sam., ix: 13.

*Injuries, &c.*—Moses speaks of broken-handed and broken-footed persons, but gives no more definite account of them than this. Luke gives an account of the cutting off an ear by one of the apostles. Jonathan had a son who, at five years of age, fell out of his nurse's arms, and was thereby made lame, (2 Sam. iv: 4.) Injuries of the testes are merely mentioned in Deut., 23, and Levit., 21, as disabling any man from performing religious duties, but no particular kind of injury is mentioned. The cutting off the privy member is also spoken of in Deut., 23. The following quotation from the 19th of Matthew is interesting in more than one particular: "There are some eunuchs, which *were so from their mother's womb;* and there are some eunuchs which were made eunuchs of men."

*Ulcers, Boils, Blains.*—Although modern language has made a difference between Ulcers and Boils, yet it is not so clear that ancient surgery did the same, and I am inclined to think that the two were confounded in olden times. It is said that

Job was afflicted " with sore boils from the sole of his foot unto his crown, and he took him a potherd (a broken piece of pottery) to scrape himself withal." The sixth plague upon the Egyptians was "a boil breaking forth with blains upon man, and upon beasts throughout the land of Egypt (B. C. 1492.")—Isaiah speaks figuratively that Israel was sorely afflicted with boils from head to foot; and in connection with it mentions the use of an ointment, but does not tell what the ointment was. It was not the Holy Ointment—in proof of which, see Exodus xxx, 32, where it is said that the Holy Ointment was never used for other than religious purposes. Isaiah recommends "a lump of figs" to be applied to a boil as a remedy, (2nd Kings xx, 7). The above ointment was in use about 700 years B. C., and was applied directly to the boil. I find four cases of ulcers mentioned in the Bible.

*Mortification.*—I find but two cases of this effect of inflamation mentioned in the Bible, although without doubt it occured very often—more often perhaps among the ancients than with us, on account of their ignorance of the true principles of treatment, upon which alone can be predicated a successful prophylactic treatment of wounds and injuries, for the prevention of mortification, and gangrenous sloughing. No minutiæ of the above cases are given.

NERVOUS DISEASES.

*Paralysis.*—There is a case of paralysis mentioned in 1st Kings xiii, which occurred in the person of the king Jeroboam, whose arm, as it is there expressed " dried up, so that he could not pull it unto him again." Luke mentions the case of a man with a withered hand, and John says there were withered persons among the multitude who stood near by the pool of Siloam, waiting for the moving of the waters." The term palsy has gotten to be nearly synonymous with paralysis, at the present day, and I think was used to signify paralysis by the Biblical authors. If this be so, then we have several other cases, recorded by the New Testament authors, which I need not mention.

*Tongue-tie* (*Anhyloglossum.*)—There is to be found in the 7th chapter of Mark the description of which corresponds well with the tongue-tie. Luke, in describing the same case, says that the man "had an impediment in his speech;" and a few verses following adds, "the string of his tongue was loosed"—which "string of his tongue" means the frenum, the cutting of which is the operation performed for the tongue-tie by the moderns.

*Devils, or Unclean Spirits.*—These two expressions are synonymous; this is proven by the 42nd verse, 9th chapter of Luke—where both terms are used, and to mean the same thing: "And as he was yet a eoming, the *devil* threw him down and tare him. And Jesus rebuked the *unclean spirit*, and healed the child," &c. In Mat. xvii, 18, there is mentioned a case of Epilepsy, in which place the term *devil* is used. In Luke, ix, 42, where the same case is mentioned and described, the expression *unclean spirit* is used. Also in Mat. xv, 22, and Mark vii, 25, one and the same case is described, and in one place the term *devil* is used, and in the other the term *uncleam spirit.* They are therefore synonymous.

The first thing now, then, that claims our attention in connection with the subject, is, what is the nature of the affection termed "being possessed with a devil?" I hold that it means simply mental or nervous disorder, and that the expression, "possessed with a devil," was used by the Evangelists generically, to signify a class of diseases—that class to which we give the name of nervous or mental disorder; in other words, that the expression "possessed with a devil," used by the New Testament authors, is synonymous with our expression nervous disease, and they are only different names for the same thing. I will now prove this—that the "possessed with a devil," were simply nervous or mental disorders;—I will then classify these diseases, and give a description of them in their proper order. In John viii, 48, it is said that the Jews accused Christ as follows: "Say we not well that thou art a Samaritan, and hast a devil?" This passage with the context would be sufficient, did we not possess better evidence, to prove that the Jews meant that Christ was a madman; he taught strange doctrines—his conduct was different from that of other men—therefore he was mad, or as they expressed it, was possessed of a devil. In John x, 20, on another occasion the Jews used the following language, which is more to our purpose: "He hath a devil, and is mad; why hear ye him?" Is not the meaning of the term devil in these passages sufficiently? To my mind they indicate plainly enough the idea attached to the expressions *devil* and *unclean spirits* by the Jews. The same idea is prominent in the 15th verse, of 17th Mat.: "Lord have mercy on my son; for he is a Lunatic and sore vexed," &c. The context shows this case to be one possessed of a devil. In the 5th chapter of Mark, and 8th of Luke, there is described the case of one possessed with a devil; and when Christ healed the disease, and permitted the devils to go into the swine-herd, and the people all came out of the city to see what had been done, "they come to Jesus, and see him that was possessed with the devil, and had the legions sitting, and clothed, and *in his right mind* (Mark v, 15). And Luke, in his viii, 35, repeats the same thing, and in the same words, thus showing that before the man who was possessed was cured, he was *not* in his right mind. I need not extend the evidence further, in proof that those who are described as being possessed with devils, had simple mental or nervous disorder. I conceive the above to be sufficient.

Thus we see that all those cases described as being "possessed with a devil," are nervous in their nature. So I will now proceed to a classification of all these cases. It is necessary, however, first to give a succinct classification of nervous disases, and then it becomes easy to classify the New Testament nervous disorders. All nervous diseases may be classified as follows: *Class* 1. Nervous diseases. *Order* 1. Encephalic nervous disease. *Order* 2. Spinal nervous disease. *Order* 3. Disease of the nerves of special sense. *Order* 4. Sympathetic nervous disease. This classification might be further divided, but this is sufficient for my purpose. To Order 1, belongs Mania, and the different varieties of mental derangement. Order 2 includes all the convulsive affections, (See Marshal Hall's investigations.) Order 3 includes all those nervous affections, which are not referable to any pathological lesion of any part of the nervous system, but in which there is a paralytic condition of some one or more of the nerves of special sense, of which Amanrosis is an example. And Order 4 includes all those diseases in which there is primarily a derangement of some one or other part of the sympathetic system of nerves. And I am frequently inclined to think that to this last Order may be referred, either directly or indirectly, nine-tenths of the diseases "to which flesh is heir"—the Diarrhæs, Dysinteries, Choleras, Albuminuria, the various exanthemata, the Fevers,* &c., &c.

Now we have but to make a slight change in the above classification, and we have a classification of the Nervous diseases of the New Testament. It is as follows: *Class* 1. Possessed with a devil. *Order* 1. Encephalic possession. *Order* 2. Spinal possession. And *Order* 3. Possession affecting some nerve of special sense. We leave out *Order* 4, because we have no disease referable to it among those of which we are now treating. To *Order* 1, as above, belongs Mania. To *Order* 2, belong the convulsions. And to *Order* 3, the affections of the senses. I will now proceed to a consideration in detail of those cases of possession by a devil which are recorded in the New Testament.

*Class* 1. *Order* 1.—In Mat. viii, Mark v, and Luke viii, we find a case described, which may be very properly located in this Order. The following is the account of it. There were two demoniacs, exceedingly fierce and dangerous, who had been bound in chains but had broken loose, and were now living among the tombs. They were naked; and bruised, and lacerated themselves, nor could any man "tame" them, and always night and day, they were in the mountains and in the tombs, crying, and cutting themselves with stones. They were deranged in mind, and had been thus afflicted a long time. These are the symptoms as we gather them from the above writers. That they were deranged in mind, is proved both by Mark and Luke. Mark says in his 5th chapter, 15th verse, that when the people from the city came out, they found the men who had been possessed "sitting, and clothed, and *in their right mind.*" And Luke in his viii, 35, repeats the same thing in the same words, and thus implies that before they were cured, they were *not* in their right mind. This taken in connection with their conduct, as given above, makes out a very clear case of Mania. (In the above cases of Mania, Matthew mentions two Maniacs—Mark and Luke but one.

*See An Inquiry into the Nature of Typhoidal Fevers, based upon a consideration of their history and pathology. By H. F. Campbell, M. D. Transactions of Amer. Med. Asso., May, 1853.

Commentators think there were two, and that Mark and Luke mention only the fiercer of the two.)

*Class* 1. *Order* 2.—The only case which I find, and which properly belongs to this Order, is described in Mat. xvii, Mark ix, and Luke ix, from which chapters I gather the following description: It occurred in the person of a male youth, was not continuous, but recurred frequently; and when the attack was upon him, he would fall into the fire or river, or on the ground, and would "wallow," and lacerate and bruise himself, and "gnash his teeth and foam," and pine away. These are the symptoms in their order of sequence. Who can fail to recognize Epilepsy in this case? The sudden falling down, wherever he might be; the wallowing or convulsive phenomena; the grinding the teeth, as modern authors call it; the foaming, which Eberle, if I recollect aright, considers pathognomonic of this affection; and finally, the pining away, which is but the somnolence succeeding a fit of Epilepsy. I think it a decided case.

*Class* 1. *Order* 3.—That there were cases in which men were possessed with devils, that were dumb or blind devils, is easily proved. See Luke xi, 14 as follows: "And he was casting out a devil, and it was dumb. And it came to pass when the devil was gone out, the dumb spake." Another dumb devil is mentioned in Mat. ix, 32. In Mat. xii, 22. there is mentioned one blind as well as dumb: "There was brought unto him one possessed with a devil, blind and dumb; and he healed him, insomuch that the blind and dumb both spake and saw." The devil in the above case of Epilepsy was exorcised, according to Mark ix, 25, in the following language: "Thou dumb and deaf spirit, I charge thee come out of him," &c. In the first of these quotations the devil is called plainly a dumb devil; in that from Mat. xii, 22, the same thing is implied; and in the last from Mark ix, 25, it is again plainly called "dumb and deaf spirit." That some of these diseases may have been organic affections of the organs or nerves of special sense, I do not deny, but, for convenience, I have arranged them all under the caption of disease of the nerves of special sense.

There is mention made of a large number of persons possessed with devils, but of which there is not sufficient description to enable us to classify them; indeed, they are merely mentioned, and we can therefore only refer to them. The above are all of which we have any definite accounts. But it is presumable that those not described might be referred to some one or other of the orders into which we have divided the New Testament nervous diseases, had we sufficient details to ascertain their identity. We can, as it is, only refer to them. They are found mentioned in the following chapters: Mat. iv, 24; Mat. viii, 16; Mat. xv, 22; Mark i, 32; Mark iii, 11; Mark vii, 25, Luke vi, 18, and Acts viii, 7.

*Fever.*—Among the punishments mentioned in 28th Deut., to be inflicted upon the Israelites for disobedience of the Commandments, it is said, among other things, that they should be smitten with a fever. This is all that we have of fever in this or any other part of the Old Testament. But we find mention made of fever again in Mark i, and Luke iv, both in reference to the same case, that of Simon's wife's mother. I am not able to determine the type of this fever, because nothing more than the mere existence of the fever is mentioned. We may deduce our conclusion however, and that is, that at least one form or type of fever existed 1451 years B. C., as well as in the year 31 of the Christian era. Josephus says that a certain king, Alexander, had a "quartan ague" for three years, and of which he finally died.

*Consumption.*—This is another disease mentioned among the punishments to be inflicted upon the Israelites for disobedience of the Commandments; but whether it means Phthisis Pulmonalis or not, I am not able to determine. I presume however that that was not the meaning, attached to it by the Divine Lawgiver.

*Dysintery*—I find one case of this disease in Acts xxviii, spoken of in the following language: "and it came to pass that the father of Publius lay sick of a fever and of a *bloody-flux.*" It is presumable that the fever was but the accompanying fever of Dysintery—which we find to exist, to a greater or less extent, in all Dysinteries.

*Dropsy.*—This disease is mentioned once, in Luke ii, but with no particulars regarding the case. This is the only mention made of dropsical effusion in all the Bible.

*Onanism.*—This disease, if it be properly a disease, is mentioned twice in the Bible—Gen. xxviii, and Levit. xv. Genesis says that a man named Onan [from whom the disease probably derived its name (?)] was directed to go in unto his brother's wife, and raise up seed unto his brother who was dead; but Onan knowing that they would not be his children, spilled his seed upon the ground. Leviticus speaks of it as follows: "if any man's seed shall go out from him." This may mean either by Onanism, nocturnal emission or spermattorrhea. The above demonstrates quite clearly, however, that Onanism existed 1725 years B. C.

*Affections of the Senses.*—Among these affections I include Dumbness, simply because it is an affection of one of sense. I find among the Biblical authors, accounts of eight cases of Blindness, one of Dumbness, one of Deafness, and one in which existed both Blindness and Dumbness. These cases are treated of more *in extenso* elsewhere, and I shall merely add that there is nothing of interest connected with them beyond the fact that one of the cases of Blindness was congenital.

PHYSIOLOGICAL DICTETICAL.

Under this caption I will include one or two cases which I cannot better classify. Luke says that Christ "sweat blood." This must have been a Pathologico-Physiological action—an unnatural action of the perspiratory emunctories of the skin, by which blood was allowed to find an exit through them. It may have been an effusion, or a something analogous to a hemorrhage, from the capillaries of the perspiratory glands, into the excretory ducts of those glands.

Jonathan, on one occasion ate a little honey, and his understanding was thereby greatly improved. He made the following remark on the occasion: "See, I pray you, how mine eyes have been enlightened, because I tasted a little of this honey."

On one occasion Daniel requested the chief of the eunuchs to allow him and his companions no meat to eat, but only "pulse to eat, and water to drink." The eunuch objected, saying that the king would be angry if Daniel should look ill. But Daniel finally prevailed upon him to allow them their preference in food for ten days; and at the expiration of that time it is said that "their countenances appeared fairer and fatter in flesh than all the children which did eat the portion of the king's meat." The pulse here spoken of, is a diet made of some of the coarser grains, such as peas, beans, &c., so that Daniel's diet during those ten days was entirely vegetable.

*Unknown Diseases.*—I have already asserted that our diseases of the present day did not have their origin with us, but are a part and parcel of the legacy left to us by former generations of men. I find, as I have already shown, a number of them spoken of in the Bible, which we can recognize, but there are others also mentioned which we cannot recognize, and which may have become extinct long before our time. These I shall now discuss.

Jehoram, one of the kings of Israel, was afflicted with an "incurable disease of the bowels," and after having the disease for two years, "his bowels fell out by reason of his sickness; so he died of sore diseases." Herod died a miserable death also, "was seized with excrutiating pains, worms bred in his putrified flesh and devoured him alive."

"Asa, king of Judea, in the 39th year of his reign was diseased in his feet, until his disease was exceeding great." He died two years after; but whether in consequence of his disease we have no means of ascertaining.

Luke, in his 7th chapter, mentions the case of a sick servant, but gives no particulars. John also, in his 5th chapter, makes mention of an "infirmity," but without telling its nature. The expressions "all manner of sickness," and "all manner of diseases among the people," which occur frequently, prove the existence of a great many diseases in that age, the names of which are not mentioned by the New Testament authors. And if we turn to the Old Testament we find mention made of a large number of diseases, by name, but without any description. I quote from Deuteronomy 28th chapter in proof of this: "The Lord shall smite thee with a consumption, and with a fever, and with an inflammation, and with an extreme burning, and with blasting, and with mildew." "And the Lord will smite thee with the botch of Egypt, and with the Emerods, and with the scab, and with the itch, whereof thou canst not be healed." "The Lord shall smite thee in the knees, and in the legs with a sore botch, which cannot be healed, from the sole of thy foot unto the top of thy head." "Then the Lord will make thy plagues wonderful, and the plagues of thy seed, even great plagues, and of long continuance, and sore sickness, and of long continuance. Moreover, He will bring upon thee all the diseases of Egypt which thou wast afraid of; and they shall cleave unto thee. Also every sickness, and every plague which is not written in the book of this law, them will the Lord bring upon thee until thou be destroyed." Here are mentioned a great many diseases with which we are not at all acquainted, at least by the names here given. We may have all these diseases among us now, but we have no means of proving it, and of identifying any or all of them with any of our diseases. The author of Deuteronomy ends by saying, that not only those diseases which are here mentioned, but also every other disease "which is not written down in the book of this law" shall be visited upon the disobedient. "Every other"—this implies a great number.

### MATERIA MEDICA AND THERAPEUTICS.

*Vomiting from fullness.*—I find that this physiological action of the stomach was known to the Hebrews, by the following from the Proverbs of Solomon: "Hast thou found honey? Eat so much as is sufficient for thee, lest thou be filled therewith and vomit it."

*Bitter water made sweet.*—During the journeyings of the Israelites, they arrived at one place where all the water was bitter and unfit to drink, from which circumstance the place derived its name Marah, which means bitterness. Moses made the water sweet and palatable by putting into them the branches of a tree, the name of which is not told.—(Exod. xv., 25.) The waters of Jericho were at one time possessed of bad qualities, but this was remedied by Elisha, simply by throwing salt into them.—(2d Kings, ii., 19-21.)

*Holy Ointment and Perfume.*—The following formulæ, are the recipes mentioned in Exodus for making these compounds, reduced to English weights and measures: Holy Ointment—℞. Pure Myrrh and Sweet Calamus, aa, 2 oz., 5 pennyweights, 15 grains, Sweet Cinnamon and Cassia, aa, 1 oz., 2 pennyweights, 18½ grains, Olive Oil, O 10. Holy Perfume.—Stacte, Onycha, Galbanum and pure Frankincense, aa, equal parts. The Calamus of the first recipe, was a shrub of Egypt, which grew about two feet high, and was very fragrant. The Stacte of the Perfume, was the purest quality of Myrrh, that which exuded from the tree without incisions. The Onycha was probably an oderiferous shell or gum. "A species of muscle is still found in the Red Sea, the shell of which, when burnt, emits a smell not unlike musk."

The Holy Ointment was never used for other than religious purposes.—(Exod. xxx., 32.) But there is an Ointment spoken of, of which we do not know the composition, used as an application to ulcers; it is mentioned in Isaiah, 1st chapter. Isaiah is speaking of "wounds, and bruises, and putrifying sores," which, says he, "were not closed, neither bound up, neither mollified with ointment." Mark uses the following language—"They anointed with oil many that were sick, and healed them." James also speaks of the "anointing with oil" in cases of sickness. See also Luke, x., 34.

*Paul's Prescription, &c.*—It is probable that Timothy either had been sick, or was in very feeble health, and in consequence, Paul advised him to "drink no longer water, but use a little wine for thy stomach's sake, and thine often infirmities"—a very good prescription for one in feeble health, or weakened by disease. Isaiah's remedy for ulcers, boils, &c., as already mentioned, was a "lump of figs." The Materia Medica of the seventh century B. C., was more extensive than any one would suppose. Jeremiah uses the following language: "In vain shalt thou use many medicines."

*Chemistry.*—The following extract shows that chemistry was not wholly unknown to the ancient world; and, it is a reasonable presumption to suppose, that if they were sufficiently acquainted with the reactions of chemical agents to be able to detect and identify the existence of lime in burnt bones, that it is equally supposable, that they were pretty extensively initiated into the mysteries of chemistry. I quote from Amos 2d—"He burned the bones of the king of Edom into lime."

I will close this section of my subject with the following condensed statement of the medicinal and non-medicinal vegetable and mineral substances mentioned in the Bible. The figures attached to the names represent the number of times the substances are mentioned. I cannot give the references, it would occupy too much space.

*Medicinal Vegetables.*—Fir (17), Pine (3), Myrtle (4), Cinnamon (3), Cassia (3), Frankincense (13), Myrrh (11), Olive (20), Fig tree (24), Pomegranate (20), Balsam or Balm (5), Almond (5), Palm or Date (15), Mandrake (2), Aloes (3), Spikenard (2),

Hyssop (1), Anise or Dill (1), Saffron (1), Oil (4), Coriander (1), Calamus (5), and Mustard (1).

*Medicinal Minerals.*—Galbanum (1), Gold (12), Silver (15), Lead (6), Tin (3), Iron (12), and Copper (see Brass, (6).

*Non-Medicinal Vegetables.*—Cedar (4), Oak (7), Cypress (1), Poplar (1), Willow (6), Shittim wood (2), Gopher wood (1), Sycamore (5), Apple tree or Citron tree (2), Vine (34), Lily (5), Rose (2), Cucumber (1), Mellons (1), Leeks (1), Onions (1), Thistles or Thorns (12), Nettles (5), Tare (1), Wheat (6), Millet (1), Barley (4), Beans (1), Lentils (4), Fitches (2), Cummin (2), Flax (3), Corn (9), (the word corn is used generically in the Bible to signify any kind of grain,) Garlic (1), Chestnut tree (2), Rue (1), Onycha (1), Mint (2), and Rye (2).

*Non-Medicinal Minerals.*—Bdelium (2).

It may be proper here to remark, that the Balm or Ointment spoken of in the Bible as the Balm of Gilead, was nothing more than simple Turpentine.

Music was also used at times as a remedial means, as we see by 1st Samuel, xvi., 16.

The Israelites were liable to be visited by various Plagues or Pestilences, as they are called in the Bible, and we are not therefore surprised, to find that they made use of various prophylactic means for preventing the development or spread of such epidemics. The means mentioned in the Bible are as follows: Dietetic rules (Levit. xi.); Separation, or, as we would call it, Quarentine; Ablutions; Anointings; and purifying clothes and houses, (Levit. xiv). A great many physicians include circumcision among the prophylactics, but without any satisfactory reason, so far as I am able to ascertain. The fact that it can prevent any one from contracting Syphilis or Gonorrhœa, is no reason for believing that it was instituted for that purpose among the Israelites. That may have been a part of the object of its institution, but we have no proof of it.

In 2d Kings, ix., 30, it is said that Jezebel painted her face. We (at least *I* cannot) cannot form any opinion as to the composition of this paint, nor as to its color, although Pareira calls it the Sesqui-Sulphuret of Antimony; but upon what grounds he bases his conclusion I am unable to say.

